# SOP: thrombolysis in ischemic stroke under oral anticoagulation therapy

**DOI:** 10.1186/s42466-022-00174-z

**Published:** 2022-02-17

**Authors:** Pawel Kermer, Peter D. Schellinger, Peter A. Ringleb, Martin Köhrmann

**Affiliations:** 1 Department of Neurology, Friesland Kliniken gGmbH, Campus Nordwest-Krankenhaus Sanderbusch, Am Gut Sanderbusch 1, 26452 Sande, Germany; 2 Department of Neurology, University Medicine Göttingen, 37075 Göttingen, Germany; 3 Department of Neurology and Neurogeriatrics, University Hospital, Johannes Wesling Klinikum/RU Bochum, Hans-Nolte-Straße 1, 32429 Minden, Germany; 4 Department of Neurology, University Hospital Heidelberg, Im Neuenheimer Feld 400, 69120 Heidelberg, Germany; 5 Department of Neurology and Center for Translational Neuro- and Behavioral Sciences (C-TNBS), University Hospital Essen, Hufelandstrasse 55, 45147 Essen, Germany

**Keywords:** Acute ischemic stroke, Oral anticoagulation, Antidote, Iv-thrombolysis, Rt-PA

## Abstract

**Introduction:**

Based on demographical trends and the expected worldwide increase in the number of individuals with atrial fibrillation, the rate of patients who are on oral anticoagulation therapy for secondary prevention of stroke rises continuously. Despite correct drug intake and good adherence to the respective medication, recurrent ischemic stroke still occurs in ~ 3% of patients. The question how to deal with such patients with regard to intravenous thrombolysis with rt-PA within the 4.5 h time window is of great relevance for daily clinical routine. However, international guidelines can be considered heterogenous or do even lack recommendations on this topic especially in light of available reversal agents. Therefore, we provide this SOP.

**Comments:**

Beyond the identification of acute stroke patients on oral anticoagulation therapy, the type of medication, time since last intake, renal function and laboratory exams as well as the availability of reversal agents have to be considered before rt-PA application and potential endovascular therapy. Treatment on a Stroke Unit or Neuro-ICU is certainly recommended in any of those patients.

**Conclusions:**

This standardized operating procedure was designed to guide stroke physicians through questions on eligibility for rt-PA treatment in patients with acute ischemic stroke who are on approved oral anticoagulation therapy thereby increasing the number of patients benefitting from thrombolysis and minimizing door-to-needle times.

## Introduction

Cardioembolic strokes in patients with atrial fibrillation (AF) represent a significant proportion of ischemic strokes [1]. Oral anticoagulation is an effective primary and secondary preventive measure in this situation [2]. Since the introduction of oral factor II antagonists (dabigatran) and factor Xa antagonists (apixaban, edoxaban, rivaroxaban), most national and international guidelines recommend treatment of AF-patients with a NOAC [2]. Despite sufficient oral anticoagulation, there is a risk of up to 3% that patients will have another ischemic stroke [3]. The American Heart Association guidelines [4] recommend systemic thrombolytic therapy in patients on a Vitamin K antagonist (VKA) and an INR ≤ 1.7 based on observational data. The addendum to the DGN/DSG guideline "Acute Therapy of Ischemic Stroke" (AWMF registry number 030–046) published already in 2015 in addition recommended that systemic thrombolytic therapy may be considered in patients on NOAC treatment if sensitive coagulation tests are normal or the patient has not taken any of these drugs in the previous 48 h with normal renal function. The recently published revision of the DGN/DSG-S2e guideline "Acute Therapy of Ischemic Stroke" did not address recanalization therapy in anticoagulated patients, so no comment is made on this issue. Thus, one might wonder if iv-thrombolysis with alteplase in patients on oral anticoagulation is safe and effective. A recently published systematic review and meta-analysis based on 366 patients neither found a significant increase in the risk of symptomatic intracerebral hemorrhage (according to the ECASS-2 definition) after systemic thrombolysis when taking a NOAC compared to systemic thrombolysis in patients taking VKA (OR 0.77; 95% CI 0.28–2.16), nor compared to patients not on oral anticoagulation (OR 0.92; 95% CI 0.33–2.55) [5]. However, these patients had not been previously treated with any of the available antidotes. Idarucizumab is an antidote for dabigatran, which is approved and has been available since 2016. In Germany, idarucizumab is approved for the treatment of adult patients, for rapid reversal of the anticoagulant effect of dabigatran when emergency surgery/urgent intervention is required or in case of life-threatening and uncontrollable bleeding. In REVERSE-AD, a total of 503 patients were enrolled: 301 in group A (uncontrolled bleeding), and 202 in group B (urgent procedure planned). The median maximum percentage reversal of dabigatran was 100% (95%CI 100–100), on the basis of either the diluted thrombin time or the ecarin clotting time. In group B, the median time to the initiation of the intended procedure was 1.6 h; periprocedural hemostasis was assessed as normal in 93.4% of the patients, mildly abnormal in 5.1%, and moderately abnormal in 1.5%. At 90 days, thrombotic events had occurred in 6.3% of the patients in group A and in 7.4% in group B, and the mortality rate was 18.8% and 18.9%, respectively. There were no serious adverse safety signals [6].

Andexanet alfa is a modified recombinant inactive form of human factor Xa developed for reversal of factor Xa inhibitors, which was evaluated in the ANNEXA-4 study [7]. 352 patients who had acute major bleeding within 18 h after administration of a factor Xa inhibitor were included. Bleeding was predominantly intracranial (64%) or gastrointestinal (26%). In patients who had received apixaban, the median anti-factor Xa activity decreased from 149.7 ng per milliliter at baseline to 11.1 ng per milliliter after the andexanet bolus (92% reduction; 95%CI, 91–93); in patients who had received rivaroxaban, the median value decreased from 211.8 ng per milliliter to 14.2 ng per milliliter (92% reduction; 95%CI 88–94). Excellent or good hemostasis occurred in 204 of 249 patients (82%) who could be evaluated. Within 30 days, death occurred in 49 patients (14%) and a thrombotic event in 34 (10%). Reduction in anti-factor Xa activity was not predictive of hemostatic efficacy overall but was modestly predictive in patients with intracranial hemorrhage. Based on this study, there is an approval in Germany for andexanet alfa in patients who have acute life-threatening or uncontrollable bleeding on apixaban or rivaroxaban. Of note, the ongoing ANNEXA-i study is recruiting up to 900 patients with intracerebral hemorrhage including such on edoxaban randomizing them either to standard treatment or andexanet alfa.

The European Heart Rhythm Association as well as the European Stroke Organisation guideline both published in 2021 recommend to consider thrombolysis in selected patients on dabigatran after reversal treatment with idarucizumab. The use of systemic thrombolysis after prior antagonization with andexanet alfa is not recommended. In addition, endovascular stroke therapy and treatment in a stroke unit or intensive care unit is recommended. It is emphasized that this recommendation is based on expert consensus and not on data from randomized trials [8, 9].

## Definitions

Acute ischemic stroke is an emergency condition requiring immediate diagnostic and therapeutic action.

Intravenous thrombolysis with rt-PA, if applicable, is the therapeutic gold standard in acute ischemic stroke. Fast decision making and application after symptom onset is mandatory to reach best functional outcome.

Oral anticoagulation with various drugs is a frequent therapy in patients with atrial fibrillation, pulmonary embolism, deep vein thrombosis and others potentially limiting therapeutic options and delaying decision making.

### First steps

- Check and secure vital functions
- Assess neurological status and obtain medical history including medication
- Check if the patient is eligible for iv-thrombolysis based on time-window and/or functional imaging.

## Comments

### Thrombolysis in patients on VKA

VKA-induced anticoagulation can rapidly be reversed by infusion of 4-factor prothombin complex concentrate (4F-PCC), however there is concern that infusion of active coagulation factor concentrates may also lead to thromboembolic complications [10] especially in a thrombogenic context of acute ischemic stroke. To date there are no systematic interventional trials on the use of any reversal agent for VKA-induced anticoagulation before thrombolysis. Several case reports as well as smaller case series report on the use of mainly 4F-PCC before IVT in VKA-treated patients with AIS. In the largest series to date 26 patients (age 77.8 ± 12.8 years) with a baseline NIHSS of 11.6 ± 5.6 points and a baseline INR of 2.3 ± 0.6 were treated using this approach. Only one patient suffered major systemic (extracranial) hemorrhage and no apparent excess of acute thrombotic complications were seen although two patients suffered recurrent ischemic stroke before restarting of anticoagulation [11]. Based on this data no firm statement guiding for the use of reversal agents before thrombolysis for VKA-treated patients seems warranted.

### Thrombolysis in patients on dabigatran

The REVERSE-AD trial [6] has shown that application of idarucizumab, a humanized Fab fragment of a monoclonal antibody which specifically binds dabigatran with very high affinity, is able to reverse anticoagulation effects of dabigatran within minutes. With inclusion of TT or dTT in routine laboratory examination in emergency rooms patients with anticoagulation effects induced by dabigatran can easily be identified. Directly following infusion of 2 × 2.5 g idarucizumab dabigatran plasma concentration dropped by more than 99% in REVERSE-AD. Since this effect lasted for more than 12 h in more than 90% of patients [6] the authors suggest that control of TT following idarucizumab infusion should not be mandatory but optional. With restoration of effective hemostasis patients can thereby regain their eligibility for in-label intravenous thrombolysis with rt-PA. Start of thrombolysis should not be delayed by laboratory parameter controls. Although, there are no available data from RCTs, a large German case series [12] as well as a summary of global cases [13] suggest, that idarucizumab-treated patients who otherwise would have been excluded from thrombolysis can benefit with a substantial NIHSS reduction of 6 to 9 points. Together with the low incidence of symptomatic intracranial hemorrhage (3.6%) and death (8.4%) which is within the range of previous studies in non-anticoagulated patients [13] this procedure may be considered effective and safe.

### Thrombolysis in patients on Xa-inhibitors

In contrast to REVERSE-AD the clinical trial program for Andexanet alfa (ANNEXA-program) focused on patients with critical bleeding and did not include patients in need for urgent medical intervention such as intravenous thrombolysis for acute ischemic stroke. Therefore, only anecdotal evidence from case descriptions is present for reversing Xa-inhibitor-associated anticoagulation. Kallmünzer et al. [14] reported a case of a 77 year-old patient on anticoagulation with apixaban who was treated with rt-PA for MCA occlusion after administering andexanet alfa. No complications were observed and thrombolysis lead to complete recanalization. However, as it was shown in the clinical trials, patients might demonstrate a remarkable rebound and even overshoot of antithrombotic-activity in the early hours after reversal with andexanet alfa. This is a matter of concern and more data are needed to evaluate anti-Xa-reversal in this context.

## Conclusions

Although updated German guidelines on the acute therapy of ischemic stroke [15] do not contain statements on the management of patients on oral anticoagulation therapy presenting with signs of ischemic stroke within the 4.5 h time-window, this is a highly relevant situation in daily clinical routine debated intensively in recent reports [16, 17]. We provide this SOP to guide stroke physicians through a work-flow resulting in easy and less time-consuming decision making. As a consequence, more patients should benefit from thrombolysis with alteplase and door-to-needle times should be reduced. This SOP is in line with ESO-guidelines [9] which recommend intravenous thrombolysis in patients on VKA when INR is ≤ 1.7. Similar to the EHRA-guidelines [8], these also contain expert consensus statements in which the combination of idarucizumab with rt-PA in patients on dabigatran is suggested over no thrombolysis [9]. Alike our flowchart, ESO-guidelines do not suggest the combination of andexanet and intravenous thrombolysis with rt-PA.
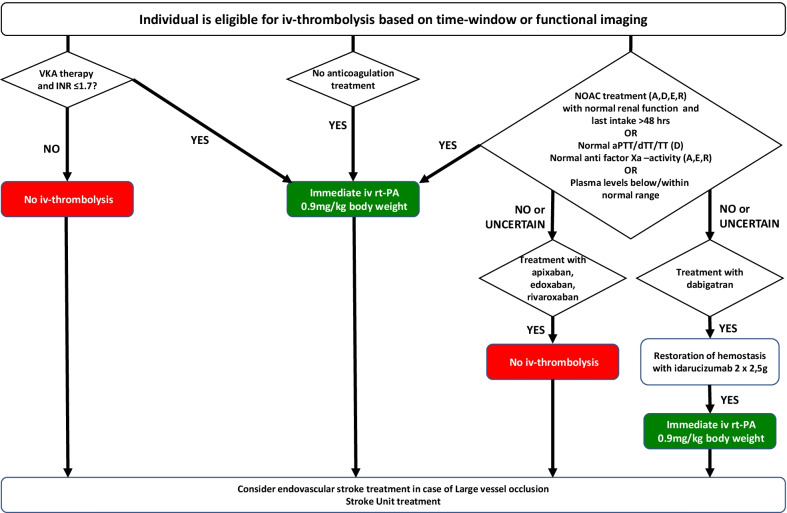


## Data Availability

Not applicable.

## Abbreviations

A: Apixaban
aPTT: Activated partial thromboplastin time
D: Dabigatran
dTT: Diluted thrombin time
E: Edoxaban
EHRA: European Heart Rhythm Association
ESO: European Stroke Organisation
EVT: Endovascular therapy
h/hrs: Hour/hours
INR: International normalized ratio
Iv: Intravenous
IVT: Intravenous thrombolysis
Kg: Kilogram
LVO: Large vessel occlusion
Mg: Milligram
MCA: Middle cerebral artery
NOAC: Non vitamin-K dependent oral anticoagulants
OAC: Oral anticoagulation
R: Rivaroxaban
RCTs: Randomized controlled trials
rt-PA: Recombinant tissue-type plasminogen activator
SOP: Standard operating procedure
TT: Thrombin time
VKA: Vitamin-K dependent anticoagulation
